# Linking health and social care data to identify teenage pregnancy outcomes for Care Experienced Young People in Fife.

**DOI:** 10.23889/ijpds.v7i3.2039

**Published:** 2022-08-25

**Authors:** Laura Hay, Lorna Watson, Peter D Donnelly

**Affiliations:** 1 University of St Andrews; 2 NHS Fife

## Objectives

Care experienced young people (CEYP) are thought to be at increased risk of experiencing teenage pregnancy but little is known about teenage pregnancy rates among CEYP in Scotland. The aim of this study was to use data linkage techniques to compare teenage pregnancy outcomes for CEYP in Fife, Scotland with that of their non-care experienced but similarly deprived peers.

## Approach

The study linked social care administrative data for 1119 females looked after by Fife Council between 1991-2015 with national health data. Probabilistic linkage was used to match CEYP demographic data (names, sex, date of birth and postcodes) to the Community Health Index (CHI) number, the unique patient identifier used in Scottish health records. The CHI number was then used to identify teenage pregnancy outcomes from national Scottish Morbidity Record datasets (SMR01 and SMR02). Outcomes for CEYP were compared with a group of non-care experienced peers from a similar socio-economic background, with three non-care experienced young people selected for every CEYP.

## Results

An acceptable match to a CHI number was achieved for 90.5% (1013/1119) of CEYP. Data analysis is ongoing and will be completed by April 2022. Outcomes being analysed include: the proportion of young people experiencing a termination of pregnancy before age 20; the proportion of young people experiencing a live birth before age 20; whether CEYP are more likely to progress a teenage pregnancy to delivery than their non-care experienced peers; antenatal behaviours such as smoking history; and postnatal outcomes such as preterm delivery.

The presentation will discuss the results. It will also discuss the challenges of linking health and social care data to identify health outcomes for CEYP.

## Conclusion

Cross-sectoral data linkage offers considerable potential for examining health outcomes for CEYP and was successfully used in this study to identify teenage pregnancy outcomes for CEYP in Fife, Scotland. Understanding outcomes will help inform prevention initiatives and address health inequalities. However, securing the necessary permissions and undertaking the linkage was a time-consuming process.

